# Supplementary material for: Longitudinal fundus imaging and its genome-wide association analysis provide evidence for a human retinal aging clock
Source: eLife. 2023 Apr 17;12:e82364. doi: 10.7554/eLife.82364 (PMC10110236; doi:10.7554/eLife.82364)
Supplement: Supplementary file 6. [file elife-82364-supp6.zip › Supplementary File 6.docx]

This tsv file is about 500MB and I was not able to upload to the system.

Please use the link below to access the file:

https://www.dropbox.com/s/jf5ukq4dmlecbe3/Supplemental%20Table%201.tsv.gz?dl=0
