# Supplementary material for: Longitudinal fundus imaging and its genome-wide association analysis provide evidence for a human retinal aging clock
Source: eLife. 2023 Apr 17;12:e82364. doi: 10.7554/eLife.82364 (PMC10110236; doi:10.7554/eLife.82364)
Supplement: Supplementary file 8. [file elife-82364-supp8.zip › Supplementary File 8.docx]

**Supplemental Table 5.** List of genes associated with eyeAgeAccel and function

| **Gene** | **P value** | **Function/disorder association** | **Reference** |
| --- | --- | --- | --- |
| SH3YL1 | 6.40E-50 | Phosphatidylcholine binding; type 2 diabetes | ^1^ |
| OCA2 | 1.90E-12 | Eye pigmentation | ^2^ |
| POC5 | 6.90E-10 | Centrosome interactions; macular degeneration | ^3^ |
| GJA3 | 1.60E-09 | Gap junction protein; cataracts | ^4^ |
| MEF2C | 1.70E-09 | Myocyte enhancement; Alzheimer's disease, muscle wasting | ^5,6^ |
| TSPAN11 | 2.20E-09 | Cell migration; cancer prognosis | ^7^ |
| NKX6-1 | 5.80E-09 | Beta cell development; cancer prognosis | ^8^ |
| GRM5 | 1.30E-08 | Metabatropic glutamate receptor; hearing loss | ^9^ |
| SLC16A1 | 2.20E-08 | Monocarboxylic acid transporter; cancer prognosis | ^10^ |
| GPR139 | 3.30E-08 | Rhodopsin family GPCR; brain metabolism with age | ^11^ |
| ARMS2 | 4.00E-08 | Eye extracellular matrix activity; macular degeneration | ^12^ |
| RAET1G | 7.70E-08 | Retinoic acid activity; cancer prognosis | ^13^ |
| ACP1 | 1.20E-07 | Phosphotyrosine protein phosphatase activity; retinopathy, diabetes, lifespan | ^14,15^ |
| ALKAL2 | 2.80E-07 | Tyrosine kinase activity; lifespan | ^16^ |
| SNTG1 | 3.00E-07 | Gamma enolase trafficking activity; Alzheimer's disease, stroke, cancer prognosis | ^17-19^ |
| BAZ2B | 3.30E-07 | Chromatin remodeling; Alzheimer's disease | ^20^ |
| GJD2 | 3.40E-07 | Gap junction activity; myopia | ^21^ |
| JAG1 | 3.70E-07 | Notch ligand activity; retinopathy, coronary artery disease | ^22,23^ |
| MYT1 | 5.20E-07 | Myelin transcription factor; age-related neuron demyelination | ^24^ |
| RASGRF1 | 5.70E-07 | Guanosine nucleotide exchange factor; myopia, neurogenesis | ^25,26^ |
| ARRDC3 | 6.20E-07 | Arrestin-related GCPR activity; Alzheimer's disease, glioma, breast cancer | ^27-29^ |
| RASSF3 | 6.60E-07 | GTP binding activity; glaucoma | ^30^ |
| COL4A3 | 7.10E-07 | Collagen activity; Alport syndrome, macular degeneration | ^31,32^ |
| DIRC3 | 8.50E-07 | Tumor suppressor activity; carcinoma prognosis | ^33^ |
| LZTFL1 | 8.80E-07 | Transcription factor activity; retinal degeneration | ^34^ |
| ATP6V0A2 | 9.30E-07 | ATPase activity; cutis laxa | ^35^ |
| GCNT3 | 9.80E-07 | Glycosyltransferase enzymatic activity; cancer prognosis | ^36^ |
| COL4A4 | 1.00E-06 | Collagen activity; Alport syndrome | ^52^ |

## Supplemental References

1. Choi GS, Min HS, Cha JJ, et al. SH3YL1 protein as a novel biomarker for diabetic nephropathy in type 2 diabetes mellitus. Nutrition, metabolism, and cardiovascular diseases : NMCD 2021; 31(2): 498-505.

2. Kamaraj B, Purohit R. Computational screening of disease-associated mutations in OCA2 gene. Cell biochemistry and biophysics 2014; 68(1): 97-109.

3. Yan Q, Ding Y, Liu Y, et al. Genome-wide analysis of disease progression in age-related macular degeneration. Human molecular genetics 2018; 27(5): 929-40.

4. Tang XJ, Shentu XC, Tang YL, Ping XY, Yu XN. The impact of GJA3 SNPs on susceptibility to age-related cataract. International journal of ophthalmology 2019; 12(6): 1008-11.

5. Xue F, Tian J, Yu C, Du H, Guo L. Type I interferon response-related microglial Mef2c deregulation at the onset of Alzheimer's pathology in 5xFAD mice. Neurobiology of disease 2021; 152: 105272.

6. Judge SM, Deyhle MR, Neyroud D, et al. MEF2c-Dependent Downregulation of Myocilin Mediates Cancer-Induced Muscle Wasting and Associates with Cachexia in Patients with Cancer. Cancer research 2020; 80(9): 1861-74.

7. Liu J, Jiang C, Xu C, et al. Identification and development of a novel invasion-related gene signature for prognosis prediction in colon adenocarcinoma. Cancer Cell Int 2021; 21(1): 101.

8. Su PH, Huang RL, Lai HC, et al. NKX6-1 mediates cancer stem-like properties and regulates sonic hedgehog signaling in leiomyosarcoma. Journal of biomedical science 2021; 28(1): 32.

9. Liu W, Johansson A, Rask-Andersen H, Rask-Andersen M. A combined genome-wide association and molecular study of age-related hearing loss in H. sapiens. BMC medicine 2021; 19(1): 302.

10. Zhang L, Song ZS, Wang ZS, Guo YL, Xu CG, Shen H. High Expression of SLC16A1 as a Biomarker to Predict Poor Prognosis of Urological Cancers. Frontiers in oncology 2021; 11: 706883.

11. Brouwer RM, Klein M, Grasby KL, et al. Genetic variants associated with longitudinal changes in brain structure across the lifespan. Nat Neurosci 2022; 25(4): 421-32.

12. Fritsche LG, Loenhardt T, Janssen A, et al. Age-related macular degeneration is associated with an unstable ARMS2 (LOC387715) mRNA. Nature genetics 2008; 40(7): 892-6.

13. Cho H, Chung JY, Kim S, et al. MICA/B and ULBP1 NKG2D ligands are independent predictors of good prognosis in cervical cancer. BMC cancer 2014; 14: 957.

14. Magrini A, Bottini N, Nicotra M, Cosmi E, Bottini E, Bergamaschi A. Smoking and the genetics of signal transduction: an association study on retinopathy in type 1 diabetes. The American journal of the medical sciences 2002; 324(6): 310-3.

15. Lucarini N, Napolioni V, Magrini A, Gloria F. The Effect of ACP(1)-ADA(1) Genetic Interaction on Human Life Span. Hum Biol 2012; 84(6): 725-33.

16. Woodling NS, Aleyakpo B, Dyson MC, et al. The neuronal receptor tyrosine kinase Alk is a target for longevity. Aging cell 2020; 19(5): e13137.

17. Velez JI, Lopera F, Creagh PK, et al. Targeting Neuroplasticity, Cardiovascular, and Cognitive-Associated Genomic Variants in Familial Alzheimer's Disease. Molecular neurobiology 2019; 56(5): 3235-43.

18. Armstrong ND, Srinivasasainagendra V, Patki A, et al. Genetic Contributors of Incident Stroke in 10,700 African Americans With Hypertension: A Meta-Analysis From the Genetics of Hypertension Associated Treatments and Reasons for Geographic and Racial Differences in Stroke Studies. Front Genet 2021; 12: 781451.

19. Meng F, Zhang L, Ren Y, Ma Q. The genomic alterations of lung adenocarcinoma and lung squamous cell carcinoma can explain the differences of their overall survival rates. Journal of cellular physiology 2019; 234(7): 10918-25.

20. Yuan J, Chang SY, Yin SG, et al. Two conserved epigenetic regulators prevent healthy ageing. Nature 2020; 579(7797): 118-22.

21. Haarman AEG, Enthoven CA, Tedja MS, et al. Phenotypic Consequences of the GJD2 Risk Genotype in Myopia Development. Investigative ophthalmology & visual science 2021; 62(10): 16.

22. Cheema MR, Stone LG, Sellar PW, et al. Long-term follow-up of a patient with JAG1-associated retinopathy. Documenta ophthalmologica Advances in ophthalmology 2021; 143(2): 237-47.

23. Kunnas T, Nikkari ST. Positive Association of the JAG1 rs1327235 Genotype with Coronary Artery Disease in Men, the Tampere Adult Population Cardiovascular Risk Study. Genet Test Mol Biomarkers 2020; 24(10): 681-4.

24. Sim FJ, Zhao C, Penderis J, Franklin RJ. The age-related decrease in CNS remyelination efficiency is attributable to an impairment of both oligodendrocyte progenitor recruitment and differentiation. The Journal of neuroscience : the official journal of the Society for Neuroscience 2002; 22(7): 2451-9.

25. Broggini T, Schnell L, Ghoochani A, et al. Plasticity Related Gene 3 (PRG3) overcomes myelin-associated growth inhibition and promotes functional recovery after spinal cord injury. Aging (Albany NY) 2016; 8(10): 2463-87.

26. Li J, Jiang D, Xiao X, et al. Evaluation of 12 myopia-associated genes in Chinese patients with high myopia. Investigative ophthalmology & visual science 2015; 56(2): 722-9.

27. Noh H, Park C, Park S, Lee YS, Cho SY, Seo H. Prediction of miRNA-mRNA associations in Alzheimer's disease mice using network topology. BMC genomics 2014; 15: 644.

28. Li N, Shi H, Hou P, et al. ARRDC3 polymorphisms may affect the risk of glioma in Chinese Han. Funct Integr Genomics 2022; 22(1): 27-33.

29. Rafiq S, Tapper W, Collins A, et al. Identification of inherited genetic variations influencing prognosis in early-onset breast cancer. Cancer research 2013; 73(6): 1883-91.

30. Fingert JH, Robin AL, Stone JL, et al. Copy number variations on chromosome 12q14 in patients with normal tension glaucoma. Human molecular genetics 2011; 20(12): 2482-94.

31. Storey H, Savige J, Sivakumar V, Abbs S, Flinter FA. COL4A3/COL4A4 mutations and features in individuals with autosomal recessive Alport syndrome. Journal of the American Society of Nephrology : JASN 2013; 24(12): 1945-54.

32. Kumaramanickavel G. Age-Related Macular Degeneration: Genetics and Biology. Asia Pac J Ophthalmol (Phila) 2016; 5(4): 229-35.

33. Shen Z, Ren W, Bai Y, et al. DIRC3 and near NABP1 genetic polymorphisms are associated laryngeal squamous cell carcinoma patient survival. Oncotarget 2016; 7(48): 79596-604.

34. Jiang J, Promchan K, Jiang H, et al. Depletion of BBS Protein LZTFL1 Affects Growth and Causes Retinal Degeneration in Mice. J Genet Genomics 2016; 43(6): 381-91.

35. Greally MT, Kalis NN, Agab W, et al. Autosomal recessive cutis laxa type 2A (ARCL2A) mimicking Ehlers-Danlos syndrome by its dermatological manifestations: report of three affected patients. American journal of medical genetics Part A 2014; 164A(5): 1245-53.

36. Fernandez LP, Sanchez-Martinez R, Vargas T, et al. The role of glycosyltransferase enzyme GCNT3 in colon and ovarian cancer prognosis and chemoresistance. Scientific reports 2018; 8(1): 8485.
